# Route Determination of Sulfur Mustard Using Nontargeted Chemical Attribution Signature Screening

**DOI:** 10.1021/acs.analchem.0c04555

**Published:** 2021-03-12

**Authors:** Karin Höjer Holmgren, Lina Mörén, Linnea Ahlinder, Andreas Larsson, Daniel Wiktelius, Rikard Norlin, Crister Åstot

**Affiliations:** Department of CBRN Defence & Security, The Swedish Defence Research Agency (FOI), Cementvägen 20, Umeå SE-901 82, Sweden

## Abstract

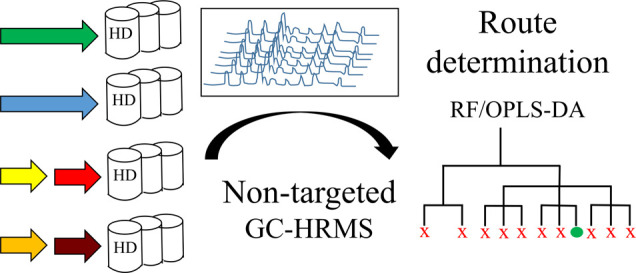

Route determination of sulfur mustard was accomplished through comprehensive nontargeted screening of chemical attribution signatures. Sulfur mustard samples prepared via 11 different synthetic routes were analyzed using gas chromatography/high-resolution mass spectrometry. A large number of compounds were detected, and multivariate data analysis of the mass spectrometric results enabled the discovery of route-specific signature profiles. The performance of two supervised machine learning algorithms for retrospective synthetic route attribution, orthogonal partial least squares discriminant analysis (OPLS-DA) and random forest (RF), were compared using external test sets. Complete classification accuracy was achieved for test set samples (2/2 and 9/9) by using classification models to resolve the one-step routes starting from ethylene and the thiodiglycol chlorination methods used in the two-step routes. Retrospective determination of initial thiodiglycol synthesis methods in sulfur mustard samples, following chlorination, was more difficult. Nevertheless, the large number of markers detected using the nontargeted methodology enabled correct assignment of 5/9 test set samples using OPLS-DA and 8/9 using RF. RF was also used to construct an 11-class model with a total classification accuracy of 10/11. The developed methods were further evaluated by classifying sulfur mustard spiked into soil and textile matrix samples. Due to matrix effects and the low spiking level (0.05% w/w), route determination was more challenging in these cases. Nevertheless, acceptable classification performance was achieved during external test set validation: chlorination methods were correctly classified for 12/18 and 11/15 in spiked soil and textile samples, respectively.

Chemical forensics is the science of attributing chemical samples to sources by analyzing their content of specific compounds or establishing links between samples based on similarities in their chemical profiles.^1^ It has become important as a tool for the attribution of alleged use of chemical warfare agents (CWA).^2−9^ The Organization for the Prohibition of Chemical Weapons (OPCW) was recently mandated to attribute the parties responsible for the use of chemical weapons in a recent armed conflict.^10^ In such investigations, chemical forensics tools can provide important data needed to link separate chemical attacks or determine how specific CWA samples were produced.^11^ Chemical forensics is also used in police investigations, for example, to determine the origin of seized drugs,^12,13^ and for fire debris investigations.^14^ Sample matching based on chemical profiling can establish a common origin of seized materials or even show that they originate from the same production batch or the same synthetic route.^3,6,15,16^ Chemical attribution signatures (CAS) may include extrinsic markers such as by-products from synthesis and chemical impurities in the starting material. An alternative approach is to examine intrinsic properties of the CWAs, such as elemental isotope ratios in the compound (s) under investigation.^17,18^

Sulfur mustard (HD) has historically been the most widely used CWA in armed conflicts,^19^ and its recent use in the Arabic Republic of Syria has been reported.^20^ HD can be synthesized via several different routes, either directly from ethylene or in a two-step process via the intermediate thiodiglycol (TDG). We have previously used a targeted method based on GC–MS analysis to identify CAS for some of these routes, enabling partial resolution of 11 production routes.^21^ By this method, all detected chemicals, not present in the blank samples, were manually included in a target library based on their mass spectra and retention indices. Both compounds identified by spectra library search and unidentified compounds were included. This method can source unknown samples correctly to single-step synthesis routes and identify the chlorination method used in two-step routes involving TDG. Unfortunately, GC–MS analyses of HD samples did not provide sufficiently detailed CAS profiles to enable discrimination between the three synthetic routes to TDG included in the study. This indicates that an analytical tool with higher sensitivity and resolution is needed to detect markers specific to particular routes of TDG synthesis. The extraction of significant CAS from large high-resolution datasets also requires an alternative strategy, possibly based on nontargeted screening.

The aim of nontargeted screening is to include as many compounds as possible by combining a broad sample extraction method with a general analytical method that can detect chemicals with diverse chemical properties.^22^ The analytical data is processed without prior assumptions about possible target compounds. Nontargeted screening has successfully been used in environmental chemistry to discover potentially toxic chemicals and investigate changes in levels of pollutants over time. For example, it was used to screen groundwater sites^23^ and sewage sludge^22^ for pollutants or to study environmental contaminants.^24^ Identification of analytes is often preferred in nontargeted screening; the level of identification can range from simply determining an exact mass to structural confirmation using reference standards.^25^

Efforts to verify alleged uses of chemical weapons can be expected to require chemical analysis of CWA in environmental samples.^26^ CWA and/or their degradation products can be extracted from contaminated samples of water, soil, or solid materials from the site of a chemical attack.^27^ There have been a few prior studies on route attribution of CWAs in relevant matrices, focusing on compounds related to production methods for Russian VX in food samples,^28,29^ acephate and CWA-related compounds extracted from dust,^30^ nerve agents extracted from indoor furnitures,^31^ and the sarin surrogate dimethyl methylphosphonate extracted from painted wallboards^32^ and office media.^5^

Chemometric data analysis and machine learning algorithms are powerful tools for the extraction of information from the high-dimensional and complex data normally generated by chemical profiling methods. Based on learning datasets, statistical models can be built in order to classify unknown samples. To be useful in court, the statistical models have to be validated and the classification results translated into a quantitative measure of evidence for or against a proposition. The use of likelihood ratio methods for evidence evaluation is established in forensic statistics,^33^ and it has also been suggested as a tool for assessment of propositions using multivariate chem-bio forensic data.^34^

The aim of the study presented here was to develop a sensitive analytical methodology based on high-resolution mass spectrometry to allow extraction of CAS suitable for discriminating between HD synthesis routes. A general nontargeted data processing method was used to enable efficient processing of large data matrices and thereby improve CAS detection.

## Experimental Procedures

### Chemicals

Ethyl acetate (99.8% purity), dichloromethane (99.8% purity), and dibenzothiophene (98% purity) were purchased from Merck, Darmstadt, Germany. Soil (Clean Sandy Loam certified reference material) from Sigma–Aldrich, MO, U.S., and a textile (unbleached cotton, 200 g/m^2^) were also used in the experiments.

### Synthesis of HD

**Figure 1:**
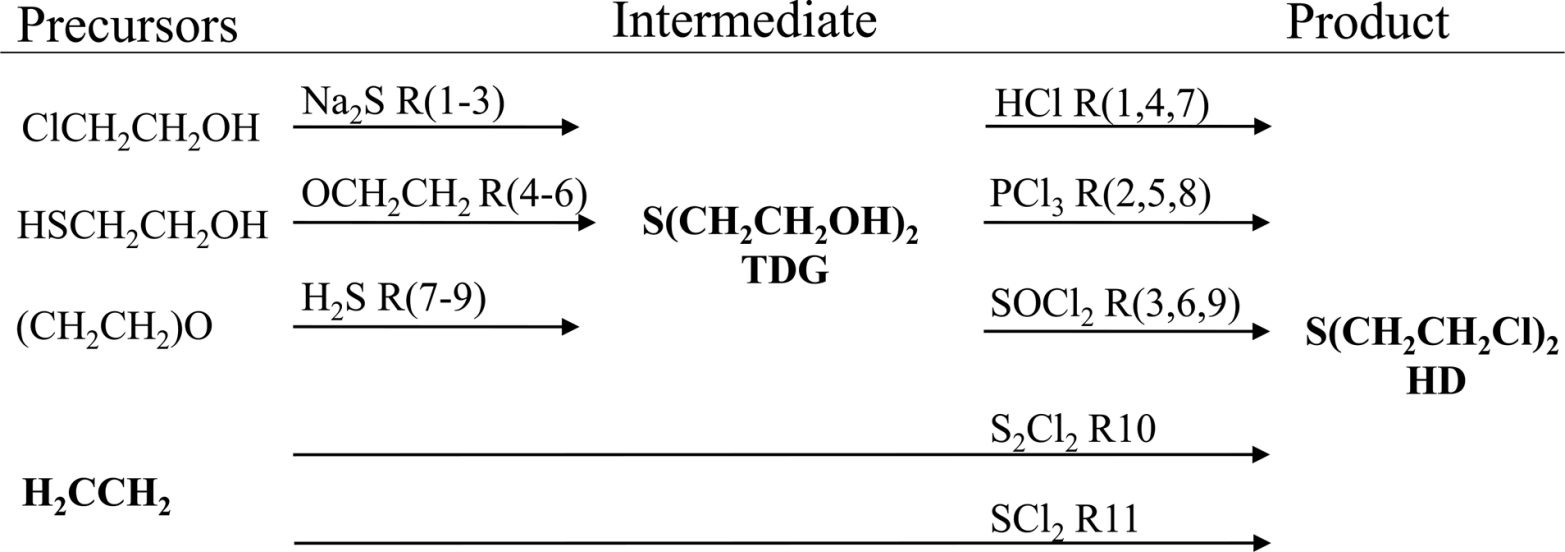
Schematic overview of HD synthesis routes. Two-step routes (R1–9) proceed via the intermediate thiodiglycol (TDG routes). HD is produced directly from ethylene in the one-step ethylene routes (R10 and R11).

Crude samples of HD representing 11 production methods (Figure 1) were prepared in house. HD was prepared via the intermediate thiodiglycol (TDG) in routes 1 to 9 (R1–R9). The TDG was produced by three methods and was subsequently transformed into HD using three chlorination protocols, resulting in nine two-step HD production methods that are collectively referred to as TDG routes. Two additional routes (R10 and R11) were included in which gaseous ethylene is directly transformed into HD. Four replicate batches of HD were synthesized by each route and were used to construct attribution models. Crude HD for a test set (one replicate of each of the TDG routes, R1–R9) was synthesized independently, approximately two years after the crude HD training set. However, test set samples for the ethylene routes were obtained from pooled training set samples representing R10 and R11. All synthesis HD batches were stored at room temperature for 1 week and then diluted to 1 mg/mL in dichloromethane, followed by storage at −20 °C.

### Sample Preparation

An overview of the samples included in this study is presented in Table 1, and an overview of the study’s workflow is shown in Figure 3. Crude HD samples, used for training and test sets, were diluted in dichloromethane to a concentration of 500 ng/μL. An internal standard, dibenzothiophene (1 ng/μL), was added to all samples and used to evaluate performance in terms of peak integration parameters and isotope ratio filters, and to enable semiquantification of CAS by TIC area comparison.

**Table 1:** Overview of Samples^a^^,^^b^^,^^c^

	crude HD samples		spiked matrix samples^a^	
synthesis routes	training set	test set	training set^b^	test set
R1	4	1	6 + 6	2 + 2
R2	4	1	6 + 6	2 + 2
R3	4	1	6 + 6	2 + 2
R4	4	1	6 + 6	2 + 2
R5	4	1	6 + 6	2 + 2
R6	4	1	6 + 6	2 + 2
R7	4	1	6 + 6	2 + 2
R8	4	1	6 + 6	2 + 2
R9	4	1	6 + 6	2 + 2
R10	4	1^c^	6 + 6	2 + 2
R11	4	1^c^	6 + 6	2 + 2

a Soil and textile matrices spiked with crude HD.

b Pooled crude samples spiked in triplicates at two occasions.

c Pooled crude sample.

All matrix training samples were spiked with pooled crude HD samples. Pooled crude HD samples representing each route (R1–R11) were prepared by mixing 250 μL of each replicate sample for the route in question. The use of pooled crude HD samples made it possible to add exactly the same solution to all six soil and textile matrix samples. Spiked matrix samples were prepared by adding 50 μL of the appropriate pooled crude HD sample (1 mg/mL) to 0.1 ± 0.01 g of soil or textile, theoretically resulting in the addition of HD to a level of 0.05% w/w. Spiked matrix training samples were prepared in triplicate on two occasions, giving six replicates for each material. The matrix test set was prepared by adding 50 μL of the crude HD test set batches (R1–R9, 1 mg/mL) to 0.1 g ± 0.01 g of soil and textile samples in duplicates. All spiked matrix samples were stored in closed glass vials at room temperature overnight. Spiked soil samples were extracted by adding 1 mL of ethyl acetate and shaking (VWR Mini Shaker, PA, U.S.) at 200 rpm for 30 min, followed by centrifugation (Allegra 25 F Beckman Coulter, Bromma, Sweden) at 2500 rpm for 3 min. Ethyl acetate was then decanted, and the extraction process was repeated once, after which the two ethyl acetate extracts were combined. Spiked textile samples were extracted by ultrasonication (Sonorex Digitec Bandlin, Berlin, Germany) in 3 mL of ethyl acetate for 10 min. The ethyl acetate phase was then removed, and the material was extracted once more, after which the two extracts were combined. The combined extracts were filtered (Chromacol PTFE 1 μm, Sigma–Aldrich, MO, U.S.) and concentrated to a volume of 50 μL under a stream of nitrogen at 40 °C. At least one blank sample of each matrix was prepared on each sample preparation occasion. The blank matrix samples were spiked with 50 μL DCM and thereafter treated identically to the spiked matrix samples.

### Chemical Analysis

Samples were analyzed with a Trace 1310 gas chromatograph coupled to an Exactive GC Orbitrap mass spectrometer (Thermo Scientific, MA, U.S.). A DB-5MS column (25 m, ID 0.25, 0.25, Agilent, CA, U.S.) was used to separate the compounds. The sample (1 μL) was injected splitless at 200 °C with helium as the carrier gas at a constant flow of 1.2 mL/min. The GC program started at 40 °C for 1 min, followed by a 10 °C/min increase to 300 °C and a hold at 300 °C for 5 min, giving a runtime of 32 min. Mass spectrometric scans were performed in the range of 30–750 m/z with a resolution of 30,000. The temperatures of the transfer line and ion source were set at 250 and 230 °C, respectively. Daily tuning and calibration ensured the quality of the mass spectrometry, and the instrument’s performance was monitored by daily analyses of QC samples.^26^ Crude HD and spiked matrix samples were analyzed in random order, and every sixth analysis run was done using a solvent blank sample. When analyzing spiked matrix samples, a solvent blank sample and a sample preparation blank were analyzed after every sixth nonblank sample.

### Nontargeted Data Processing

The chromatographic data was processed by peak detection, retention time alignment, and peak integration followed by isotope ratio filtering. This resulted in a processed dataset with peak areas from extracted ions at specific retention times corresponding to different compounds. Data processing was done in Tracefinder (version 4.1, Thermo Scientific, MA, U.S.) using the analysis mode for unknown screening, which enables nontargeted screening of data. Peak picking was done with the deconvolution plugin (version 1.3, Thermo Scientific, MA, U.S.). The retention time alignment window was set to 10 s, the accurate mass tolerance to 10 ppm, the signal-to-noise (s/n) threshold to 5, the total ion-chromatogram intensity threshold to 500,000, the ion overlap window to 90–99%, and the response threshold to 10,000.

The extracted peaks were automatically time-aligned and integrated in the unknown screening view using the Avalon detection algorithm and the nearest RT detection method with seven smoothing points. Data representing compounds present in blank samples were manually removed from each crude HD dataset. The datasets for spiked soil and textile samples were processed and manually merged after removing peaks found in blank samples. The variation in the spiked matrix data was higher than in the crude HD data, so the *m*/*z* deviation threshold was increased to 0.01 to permit merging.

### Isotope ratio filters

Data analysis was done both with and without isotope ratio filtration using sulfur and/or chlorine isotope filters, 1.9958 ± 10 ppm and 1.99705 ± 10 ppm, respectively. The internal standard was used as a control compound to ensure that peak detection was performed correctly and the sulfur isotope ratio filter settings were appropriate. Filtration was done to extract all peaks corresponding to analytes containing chlorine, sulfur, or both. It thus removed all other peaks, including matrix-associated peaks irrelevant to HD route classification. Mass defect filters have previously been used to select for metabolites based on their specific isotope ratios.^35^

### Machine Learning

Data from the crude HD samples were initially analyzed by principal component analysis (PCA) to get an overview of the variation in the data and detect potential outliers. Subsequent analyses were performed in parallel using two machine learning algorithms: orthogonal partial least squares discriminant analysis (OPLS-DA)^36^ and random forest (RF),^37^ which were performed in Simca (version 15, Sartorius Stedim Biotech, Germany) and the R software (R^38^ version 4.0.3 with a random forest 4.6–14^39^ package), respectively.

#### OPLS-DA

**Figure 2:**
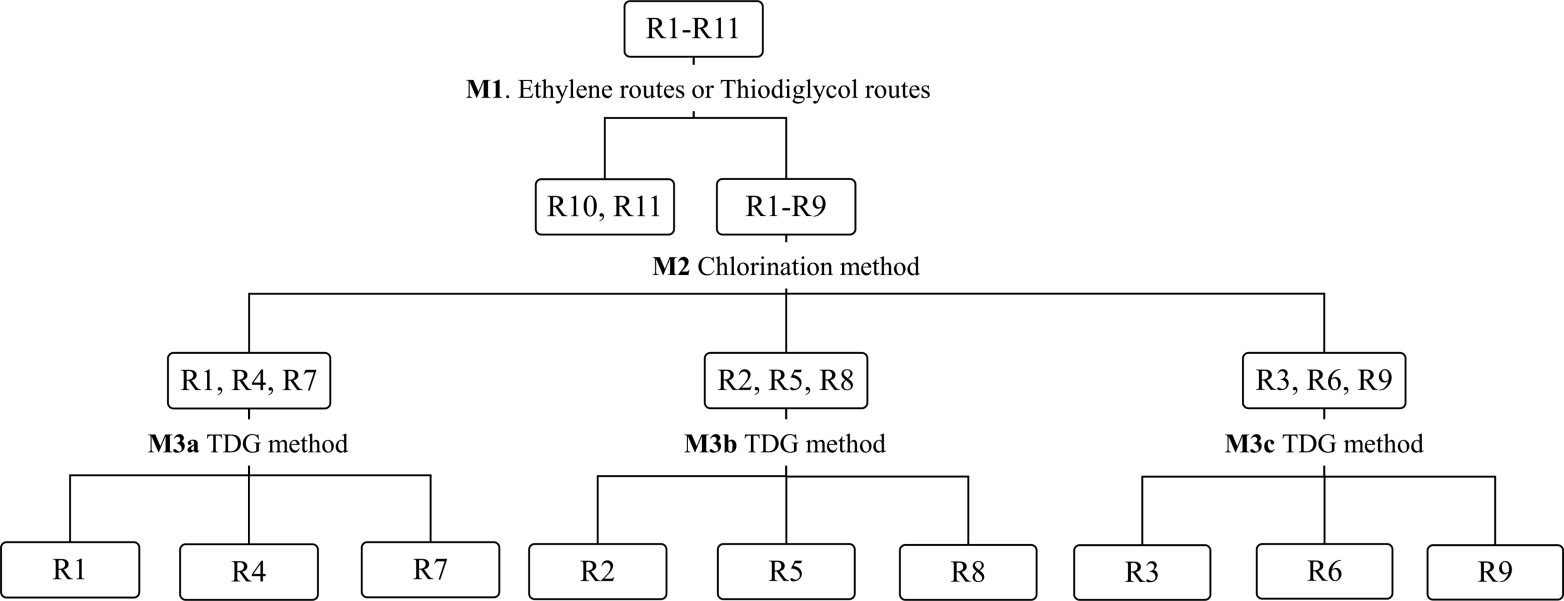
Hierarchical decision tree where the first model (M1) differentiates between ethylene route and TDG route samples. The second model (M2) differentiates between the three chlorination methods, while M3a–M3c differentiate between methods of TDG synthesis. This model tree was applied to the datasets for the crude HD samples and the spiked matrix samples

Prior to analysis, the data was normalized by total area normalization, log-transformed, and scaled to unit variance. The data was too complex to allow classification of 11 routes with a single OPLS-DA model; full classification required a hierarchic decision tree guided by multiple OPLS-DA models. A similar approach was previously used to classify production routes of the nerve agent Russian VX, based on data for spiked food samples.^28^ The hierarchic decision tree method (Figure 2) first uses a classification model, M1, which distinguishes between the TDG routes (R1–R9) and the ethylene routes (R10 and R11). A second classification model, M2, was constructed to classify HD samples attributed to the TDG two-step routes (by M1) to the method of chlorination used in their synthesis, i.e., to attribute samples to one of the groups R(1, 4, 7), R(2, 5, 8), or R(3, 6, 9). Finally, data from HD samples sharing the same chlorination method was modeled to classify them based on the method used to synthesize TDG (M3a–c). Five models were constructed for the crude HD samples and another five for the spiked matrix samples. The OPLS-DA models were evaluated by cross-validation with all sample replicates included in the same cross-validation group to avoid overfitting. The classification models were also assessed in terms of their numbers of latent variables (OPLS components) and using three measures of performance: the variation in the data matrix explained by the model (R^2^X), the variation in the response matrix explained by the model (R^2^Y), and the variation in the response matrix predicted by the model (Q^2^). CAS important for class separation were identified by their predictive variable importance in projection (VIP_predictive_) values.^40^

#### RF

The data was normalized by total area normalization. RF can handle complex datasets, so a classification model with 11 classes was constructed. The number of trees was set to 10,000 in all RF models, and the number of randomly sampled variables was optimized by comparing the out of bag (OOB)-estimated error rates for preliminary RF models with varying numbers of randomly sampled variables. The OOB-estimated error rate is the mean of the errors for each training set sample, calculated from decision trees generated while excluding the sample in question from the bootstrap sample. The number of randomly sampled variables in the final classification models is ranged from 3 to 75. To allow direct performance comparison with OPLS-DA, RF models were also used together with the hierarchical decision tree. In RF, CAS important for class separation were identified by considering their Gini impurity.^37,41^ The Gini impurity metric measures the purity of the classification tree nodes; variables with lower Gini values are more important in RF models.

### Prediction performance

All RF and OPLS-DA models were validated using corresponding HD test sets.

## Results and Discussion

**Figure 3:**
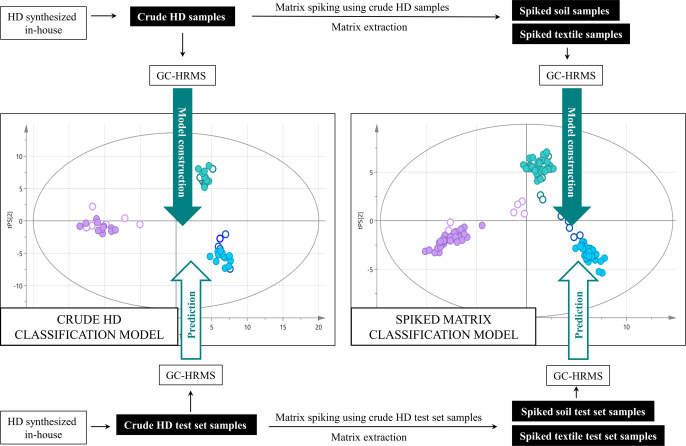
Flowchart illustrating sample use and workflow.

Attribution of chemical samples to the production method is based on CAS profiling, i.e., identification and analysis of trace components that are diagnostic for specific production conditions or starting materials used. We evaluated a nontargeted GC–HRMS method for the analysis of crude HD samples and the scope for using the data generated to build classification models (Figure 3, top left).

The models’ performance was first tested by classifying test set samples originating from different synthetic batches of crude HD (Figure 3, bottom left), This procedure was repeated on soil and textile matrix samples, spiked with crude HD (Figure 3, right). The large number of compounds detected in this study illustrated the advantages of GC–HRMS and facilitated the detection of CAS related to the 11 HD routes. A total of 2713 compounds were detected in the crude HD samples, as compared to 103 compounds using targeted GC–MS.^21^ In addition, the s/n of the 103 previously detected compounds was significantly improved by using HRMS. Another advantage of HRMS is the ability to apply isotope ratio filtration. This facilitated the extraction of relevant CAS; we expected that the most useful CAS in this context would be chlorinated or sulfur-containing compounds. Isotope filtration reduced the number of compounds to consider and improved the quality of the OPLS-DA classification models (data not shown) and was thus applied throughout the study.

### Crude HD Samples: Route Attribution through Nontargeted HRMS

The isotope ratio filtered data contained 714 potential CAS whose normalized peak areas were modeled in parallel using two machine learning algorithms, OPLS-DA and RF. The nontargeted method gave classification models of high quality (M1_crude_ and M2_crude_, Table 2). Both OPLS-DA and RF successfully distinguished ethylene routes (R10 and R11) from TDG routes (R1–R9) and predicted the chlorination methods of TDG samples with 100% classification accuracy (Table 2). These results are consistent with previous findings.^21^ A significant advantage of nontargeted data processing in this context is that there is no need to create a target library of the detected compounds for each route.

**Table 2:** Characteristics of Classification Models and Correctly Predicted Crude Test Set Samples^a^

			OPLS-DA					RF	
classification model	attribution capacity	class	comp.^a^	R^2^X _(cum)_	R^2^Y _(cum)_	Q^2^_(cum)_	prediction	OOB error (%)	prediction
M1_crude_	ethylene or TDG routes	R(1–9)	1 + 2 + 0	0.72	0.99	0.95	9/9	2.3	9/9
		R(10, 11)					2/2		2/2
M2_crude_	chlorination methods	R(1, 4, 7)	2 + 2 + 0	0.61	0.98	0.95	3/3		3/3
		R(2, 5, 8)					3/3	0.0	3/3
		R(3, 6, 9)					3/3		3/3
M3a _crude_	TDG synthesis methods of R(1, 4, 7) samples	R1	2 + 2 + 0	0.70	0.98	0.90	1/1		1/1
		R4					0/1	33.3	1/1
		R7					0/1		1/1
M3b_crude_	TDG synthesis methods of R(2, 5, 8) samples	R2	2 + 1 + 0	0.63	0.98	0.88	1/1		1/1
		R5					1/1	16.7	1/1
		R8					0/1		1/1
M3c_crude_	TDG synthesis methods of R(3, 6, 9) samples	R3	2 + 1 + 0	0.55	0.97	0.91	1/1		1/1
		R6					0/1	8.3	0/1
		R9					1/1		1/1

a Comp. shows the number of components (x/y joint predictive variation + variation in x orthogonal to y + variation in y orthogonal to x) included in each model.

### Crude HD Samples: TDG Attribution through Nontargeted HRMS

Synthesis routes R1 to R9 involve the intermediate TDG, which can be produced by three different methods (Figure 1). It is challenging to classify the method of TDG synthesis in crude HD samples because the TDG-related CAS are present at levels of <100 pg./μL, approximately 10–100 times lower than those indicative of the chlorination method. This is probably due to the high overall purity of the TDG produced (89–99.5%), and the majority of the CAS have been transformed during the chlorination step. The improved detection of CAS by nontargeted HRMS significantly increased the scope for TDG attribution. Both OPLS-DA and RF models could differentiate the TDG synthesis methods based on 714 potential CAS. The external validation results showed that classification models M3a–c_crude_ successfully classified TDG synthesis methods in HD samples (OPLS-DA, 56%; RF; 89%, Table 2), showing that the nontargeted HRMS method detected sufficient TDG-related CAS to enable comprehensive route resolution.

### CAS Determination and Compound Identity

Chemical identification of CAS is important for understanding their formation during HD production and for verifying that relevant CAS were extracted during the analysis. CAS can be identified in several ways, for example, by searching for specific compounds expected to be present based on prior knowledge, by visual comparison of detected compounds in samples, or by looking at variables found to be important in the classification models. The first two targeted approaches have limitations that restrict the amount of CAS information that can be extracted from the sample data. In this nontargeted work, we identified key CAS that were important variables in the route classification models. While some CAS were clearly associated with the chemistry of HD production, others had no obvious origin. However, evaluations of the effects of different variables on classification model performance clearly showed that successful attribution depended on complete CAS profiles rather than individual compounds (Figure S1, Table S1, S2 and S3).

The CAS found to be related to the chlorination method in two-step routes were largely identical to those previously found by GC–MS.^21^ PCl_3_ is used for chlorination in R2, R5, and R8, and gave rise to phosphorous-containing cyclic adducts, notably those with ID numbers M2_crude__11 and M2_crude__14 (Table S2). Chlorination with SOCl_2_ (R3, 6, and 9) produced HD containing chlorinated derivatives of amylene, a stabilizer in the dichloromethane used as the reaction solvent. Chlorination with HCl (R1, 4, and 7) yielded few CAS of low intensity. CAS associated with routes 10 and 11 included polysulfides and vinyl chlorides. Dithiane (ID number M3_crude__6, Table S3) is a CAS related to TDG synthesis. Most of the compounds relevant to TDG synthesis method attribution using models M3a_crude_, M3b_crude_, and M3c_crude_ could not be identified due to their low abundance (Table S3).

### Spiked Matrix Samples: HD Route Attribution through Nontargeted HRMS

**Figure 4:**
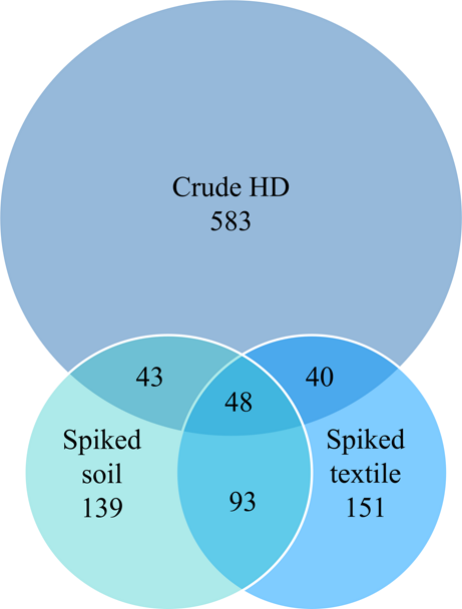
Distribution of the 1097 potential CAS found in crude HD and spiked matrices.

Because chemical forensic investigations of incidents involving chemical warfare agents may require analysis of samples taken from the environment, we also investigated the extraction of HD-associated CAS from spiked matrix samples. Route determination based on analysis of CWAs in environmental matrix samples may be complicated by interactions with the sample matrix and/or degradation of the chemicals that constitute the CAS. A low spiking level was chosen to further challenge the analytical method and classification models. Thus, 0.05% w/w of the HD produced by the various routes was spiked to the matrix samples, resulting in CAS concentrations in the range of 0.05–50 ppm. As shown in Figure S2, the most abundant peaks in the chromatograms, aside from the HD peak, originated from the matrix. The use of isotope ratio filters was essential when processing data for these samples because it extracted information on compounds containing chlorine and/or sulfur from the complex HRMS data. Despite the low spiking level, CAS profiles related to specific routes were detected in spiked matrix samples. However, there were large differences between the CAS profiles of crude HD and spiked matrix samples (Figure 4,) and the crude classifications models could not correctly classify spiked matrix samples. HD and many of the synthesis by-products are clearly highly reactive towards several matrix components, which dramatically altered the CAS attribution profiles. Only 48 of the 714 potential CAS in crude HD were chemically stable in both spiked matrices, and new potential CAS were formed in spiked soil and textile, respectively (Figure 4). The 48 CAS were not equally distributed between routes and most of them were not important for route separation. Although the CAS profiles of spiked soil and textile samples differed, sufficient common CAS were found to enable the construction of matrix models using the combined soil and textile data. Highly significant classification models could be created for the separation of ethylene routes and TDG routes, and for discriminating between chlorination methods, using either RF or OPLS-DA (Table 3). The quality (R^2^X, R^2^Y, Q^2^, and OOB) of spiked matrix models 1 and 2 (M1_matrix_ and M2_matrix_) was comparable to the corresponding crude HD models (M1_crude_ and M2_crude_, Tables 2 and 3). The chosen analytical technique and peak picking method thus made it possible to extract CAS from spiked matrix data despite the low spiking level. The most important variables in the RF and OPLS-DA M2_matrix_ models were related to PCl_3_ chlorination (R2, 5, and 8), with a few relating to SOCl_2_ chlorination (R3, 6, and 9), as shown in Table S4. Of the 12 most important variables, only one was related to HCl chlorination. The chlorination method was correctly predicted in 70% of the spiked matrix test set samples independent of classification algorithm (Table 3). Adequate classification models for predicting TDG synthesis methods could be built, but their prediction performance was slightly lower than the corresponding M3a–c_crude_ models. Spiked matrix test sets representing R7, R8, and R9 were easiest to predict, and the number of correct predictions in spiked soil samples was equal to that in textile samples, indicating that the classification models handled both matrices equally well.

**Table 3:** Classification Model Characteristics and Correctly Predicted Test Set Matrix Samples^a^^,^^b^

			OPLS-DA						RF		
classification model	attribution capacity	class	comp.^a^	*R*^2^X _(cum)_	*R*^2^Y _(cum)_	*Q*^2^_(cum)_	pred. soil	pred. textile	OOB error (%)	pred. soil	pred. textile
M1_matrix_	ethylene or TDG routes	R(10, 11)	1 + 2 + 0	0.45	0.99	0.97	-	-	0	-	-
		R(1–9)									
M2_matrix_	chlorination methods	R(1, 4, 7)	2 + 3 + 0	0.46	0.97	0.8	5/6	4/4^b^	0	5/6	4/4^b^
		R(2, 5, 8)					5/6	5/5^b^		5/6	5/5^b^
		R(3, 6, 9)					2/6	2/6		2/6	2/6
M3a_matrix_	TDG synthesis methods of R(1, 4, 7) samples	R1	2 + 2 + 0	0.75	0.93	0.66	2/2	0/1	2.9	2/2	1/1
		R4					0/2	0/1		0/2	0/1
		R7					2/2	2/2		2/2	2/2
M3b_matrix_	TDG synthesis methods of R(2, 5, 8) samples	R2	2 + 3 + 0	0.80	0.97	0.91	2/2	1/1	2.8	0/2	0/1
		R5					2/2	2/2		2/2	2/2
		R8					2/2	2/2		2/2	2/2
M3c_matrix_	TDG synthesis methods of R(3, 6, 9) samples	R3	2 + 0 + 0	0.73	0.64	0.26	0/2	0/2	0	0/2	0/2
		R6					2/2	2/2		2/2	2/2
		R9					2/2	0/2		2/2	2/2

a Comp. shows the number of components (x/y joint predictive variation + variation in x orthogonal to y + variation in y orthogonal to x) included in each model.

b In the initial PCA model, two outliers were detected among the training set samples and three in the test set and thus excluded from further analysis.

### Comparison of Classification Tools

The parallel use of OPLS-DA and RF in this study allowed us to benchmark the performance of the two classification tools. When the multimodel hierarchical decision tree (Figure 2) was used, the two methods achieved very similar predictive accuracy (Table 2). Many of the CAS important for separation by OPLS-DA and RF were identical, especially in M2_crude_ (Table S2). However, since RF is a tree-based algorithm, it does not require the use of such a decision tree; the tree was only used with RF models to permit detailed performance comparison with OPLS-DA. RF could also be used to resolve all of the classes in a single model; accordingly, 11-class models were constructed for both crude samples (M4_crude_) and matrix samples (M4_matrix_). The M4_crude_ model outperformed the OPLS-DA decision tree models for crude samples, with 10/11 correct classifications (Tables 2 and 4).

**Table 4:** Prediction Performance of 11-Class RF Models for Crude HD (M4_crude_) and Spiked Matrices (M4_matrix_)^a^

		test set samples		
model	OOB error (%)	crude HD	spiked soil	spiked textile
M4_crude_	20.5	**10**/11^a^	-	-
M4_matrix_	0.8	-	**8**/18^a^	**10**/15^a^

a Number of **correct**/total predictions.

Conversely, the 11-class RF model for spiked matrix samples (M4_matrix_) performed less well than the hierarchical decision tree models generated using both RF and OPLS-DA. In the test set validation of model M4_matrix_ (Table 4), 18 samples out of 33 were classified correctly.

While the performance differences between the classification methods were small, they differed in their handling of misclassifications: OPLS-DA models assign samples that cannot be classified as not belonging to any class (no class-category in Table 5), whereas RF models force samples into one class. This property was shown when relevant CAS could not be detected in matrix samples spiked with low levels of HD and the samples were incorrectly assigned to the HCl chlorination routes by RF (Table 5). The CAS profile associated with HCl chlorination (R1, 4, 7) is sparse, making it sensible to incorrect classifications of samples with low CAS levels. This RF misclassification problem also affected the 11-class models.

**Table 5:** Prediction Performance of OPLS-DA and RF Classification Models for Chlorination Methods in Spiked Matrices (M2_matrix_)^a^

	predicted class						
	OPLS-DA				RF		
true class	R1, 4, 7	R2, 5, 8	R3, 6, 9	no class	R1, 4, 7	R2, 5, 8	R3, 6, 9
R1, 4, 7	**9**^a^			1	**9**^a^	1	
R2, 5, 8	1	**7**^a^		3	1	**10**^a^	
R3, 6, 9	1		**4**^a^	7	8		**4**^a^

a Number of **correct** predictions.

### Future Prospects

The data presented here would preferably be used to develop a robust and efficient targeted method using the CAS database. The nontargeted approach is not readily applicable to authentic samples because it requires reference data acquired under identical instrumental conditions (e.g., analyzed in the same sample batch). Such data would be difficult to acquire because the composition of the HD reference samples is not necessarily stable over time. A CAS HRMS library would enable easy processing of new samples and could also be shared between laboratories. CAS for inclusion in such a database could be selected based on their relevance for route classification. Some (48/714) of the CAS from crude HD and spiked matrix samples were found in all three datasets, but many variables were matrix-specific (Figure 4). This difficulty could be overcome by using separate target libraries for crude and spiked matrix samples.

Bayesian statistics is a widely accepted forensic statistic framework^33^ used in order to support court decisions. It could be applied to the classification models presented above, in which estimated assignment probabilities are used, together with prior information, to calculate likelihood ratios of competing propositions. The outcome (i.e., the likelihood ratio) can then be communicated to court hearings in a transparent and scientific sound way. The methods for such calculations based on multivariate data are currently under development.^14,34^

## Conclusions

We successfully developed a nontargeted method for attribution of crude HD and spiked matrix samples to specific synthesis routes using HRMS data. The nontargeted approach enabled efficient processing of large numbers of CAS, and the high recovery of CAS facilitated the generation of significant HD route classification models using two independent classification methods, OPLS-DA and RF. The two classification methods achieved similar classification accuracy. Classification performance was very good for crude HD samples but somewhat lower for spiked matrix samples due to matrix effects. Route determination of the spiked matrices samples was made difficult due to the low spiking level. Real-world samples involving HD, may have higher concentrations, making the classification of routes easier. Overall, our results show that nontargeted methods can be valuable tools for CAS screening in chemical forensics, and that a CAS library could be a powerful tool in future investigations into alleged uses of CWA.

## Supporting Information Available

(Table S1) Most important variables in M1_crude_ models; (Table S2) most important variables in M2_crude_ models; (Table S3) most important variables in M3_crude_ models; (Table S4) most important variables in M2_matrix_ models; (Figure S1) HD synthesis descriptions and representative CAS; (Figure S2) total ion chromatogram of blank matrix samples and spiked matrix samples (PDF)
