# Supplementary material for: Route Determination of Sulfur Mustard Using Nontargeted Chemical Attribution Signature Screening
Source: Anal Chem. 2021 Mar 12;93(11):4850–8. doi: 10.1021/acs.analchem.0c04555 (PMC8041246; doi:10.1021/acs.analchem.0c04555)
Supplement: Supplementary file 1 — ac0c04555_si_001.pdf [file ac0c04555_si_001.pdf]

SUPPORTING INFORMATION

Route determination of sulfur mustard using non-targeted chemical attribution signature screening

Karin Höjer Holmgren, Lina Mörén, Linnea Ahlinder, Andreas Larsson, Daniel Wiktelius, Rikard Norlin and Crister Åstot*

astot@foi.se

Department of CBRN Defence & Security, The Swedish Defence Research Agency (FOI), Cementvägen 20, Umeå, SE-901 82, Sweden

Table of Contents

Tables

Table S1……………………………………………………………………………S2

Table S2……………………………………………………………………………S2

Table S3……………………………………………………………………………S3

Table S4……………………………………………………………………………S4

Figures

Figure S1……………………………………………………………………………S4

Figure S2……………………………………………………………………………S5

Table S1. Most important variables in M1$_{crude}$ models separating ethylene R(10-11) or TDG routes R(1-9) in crude HD samples listed according to retention time.

ID number	Peak ID Ret. time and m/z	Classification method	Molecular formula	CAS number
M1$_{crude}$_1	peak @ 9.05 102.01	RF	C_3H_6ClS (T) (Δ=1.0)	
M1$_{crude}$_2	peak @ 10.36 75.94	RF	Uid	
M1$_{crude}$_3	peak @ 9.51 155.96	RF and OPLS-DA	$C_4H_6Cl_2S$ (C) #	873408-12-9
M1$_{crude}$_4	peak @ 10.11 119.98	RF	Uid	
M1$_{crude}$_5	peak @ 11.76 140.93	RF and OPLS-DA	$C_4H_5Cl_3S$ (T) (Δ =0.5)	
M1$_{crude}$_6	peak @ 11.90 118.97	OPLS-DA	Uid	
M1$_{crude}$_7	peak @ 12.20 157.97	RF	Uid	
M1$_{crud}$ _8	peak @ 12.38 153.90*	RF and OPLS-DA	$C_4H_6Cl_2S_2$ (T) (Δ =0.04)	
M1$_{crude}$_9	peak @ 12.58 78.99	RF and OPLS-DA	$C_4H_5Cl_3S$ (C) #	153628-01-4
M1$_{crude}$_10	peak @ 12.74 78.99	OPLS-DA	$C_4H_5Cl_3S$ (C) (stereoisomer) #	153628-01-4
M1$_{crude}$_11	peak @ 17.00 180.99	RF	Uid	
M1$_{crude}$_12	peak @ 18.33 137.94	RF and OPLS-DA	Uid	
M1$_{crude}$_13	peak @ 18.51 251.92	RF and OPLS-DA	$C_6H_9Cl_3S_2$ (C) #	
M1$_{crude}$_14	peak @ 18.92 183.90	OPLS-DA	$C_6H_8Cl_4S_2$ (C) #	
M1$_{crude}$_15	peak @ 19.16 60.00	RF	$C_4H_8Cl_2S_4$ (C) #	90586-78-0
M1$_{crude}$_16	peak @ 19.47 257.85	RF and OPLS-DA	S_8 #	10544-50-0
M1$_{crude}$_17	peak @ 19.65 192.92	RF and OPLS-DA	Uid	
M1$_{crude}$_18	peak @ 19.65 222.88	RF and OPLS-DA	$C_6H_8S_2Cl_4$ (C) #	
M1$_{crude}$_19	peak @ 20.42 182.92	OPLS-DA	$C_6H_8Cl_2S_3$ (T) (Δ =0.1)	
M1$_{crude}$_20	peak @ 20.88 151.95	OPLS-DA	$C_6H_9Cl_3S_3$ (T) (Δ =0.1) #	
M1$_{crude}$_21	peak @ 21.05 283.89	RF and OPLS-DA	$C_6H_9Cl_3S_3$ (T) (Δ =0.1) (stereoisomer) #	
M1$_{crude}$_22	peak @ 21.24 207.95	OPLS-DA	Uid	

*= found in matrix models, T=tentative molecular formula, calculated by HRMS data, C= molecular formula was confirmed by GC-HRMS, # = compound detected in LR GC-MS[1], Uid= unidentified compound. Δ= ppm deviation of calculated value from theoretical elemental composition.

Table S2. The most important variables in M2$_{crude}$ models separating the chlorination methods of TDG routes (R(1,4,7), R(2,5,8) and R(3,6,9)) in crude HD samples, listed according to retention time.

ID number	Peak ID Ret. time and m/z	Classification method	Correlated to	Molecular formula
M2$_{crude}$_1	peak @ 9.58 68.06	RF and OPLS-DA	R(3,6,9)	$C_7H_{13}ClS$ (C) #
M2$_{crude}$_2	peak @ 11.57 137.02	RF and OPLS-DA	R(1,4,7)	Uid
M2$_{crude}$_3	peak @ 11.59 203.92	RF and OPLS-DA	R(1,4,7)	Uid
M2$_{crude}$_4	peak @ 12.51 91.03	OPLS-DA	R(3,6,9)	Uid
M2$_{crude}$_5	peak @ 12.62 345.02	OPLS-DA	R(2,5,8)	$C_{10}H_{18}O_7PS_2$ (T) (Δ = -0.1) #
M2$_{crude}$_6	peak @ 12.65 137.02	RF and OPLS-DA	R(3,6,9)	$C_7H_{14}Cl_2S$ (C) #
M2$_{crude}$_7	peak @ 12.85 69.07	OPLS-DA	R(3,6,9)	Uid
M2$_{crude}$_8	peak @ 13.39 93.96	RF and OPLS-DA	R(2,5,8), R(3,6,9)	$C_4H_9OClS_2$ (T) (Δ = 1.2)
M2$_{crude}$_9	peak @ 13.40 212.99	RF and OPLS-DA	R(1,4,7)	Uid
M2$_{crude}$_10	peak @ 13.43 189.94	RF and OPLS-DA	R(2,5,8), R(3,6,9)	$C_4H_8Cl_2S_2$ (C) #
M2$_{crude}$_11	peak @ 14.04 85.01	RF and OPLS-DA	R(2,5,8)	$C_4H_9O_3PS$ (C) #
M2$_{crude}$_12	peak @ 14.90 101.04	OPLS-DA	R(2,5,8)	$C_5H_{10}O_2ClPS$ (T) (Δ = 0.3)
M2$_{crude}$_13	peak @ 15.66 137.02*	RF and OPLS-DA	R(2,5,8)	$C_6H_9Cl_3S$ (T) (Δ = -0.2) #
M2$_{crude}$_14	peak @ 16.99 85.01*	RF and OPLS-DA	R(2,5,8)	$C_4H_9O_3PS_2$ (C) #
M2$_{crude}$_15	peak @ 20.98 223.89	OPLS-DA	R(2,5,8)	Uid

*= found in matrix models, T=tentative molecular formula, calculated by HRMS data, C= molecular formula was confirmed by GC-HRMS, # = compound detected in LR GC-MS[1], Uid= unidentified compound. Δ= ppm deviation of calculated value from theoretical elemental composition.

[1] Holmgren, K. H.; Hok, S.; Magnusson, R.; Larsson, A.; Astot, C.; Koester, C.; Mew, D.; Vu, A. K.; Alcaraz, A.; Williams, A. M.; Norlin, R.; Wiktelius, D., Synthesis route attribution of sulfur mustard by multivariate data analysis of chemical signatures. *Talanta* **2018**, 186, 615-621.

Table S3. Most important variables in $M3_{crude}$ models separating TDG production methods in crude HD samples, listed according to retention time.

ID number	Peak ID Ret. time and m/z	Model	M3a, M3b or M3c	Molecular formula	CAS number
$M3_{crude}$_1	peak @ 5.10 77.98	RF and OPLS-DA	M3c		
$M3_{crude}$_2	peak @ 5.16 49.98	RF and OPLS-DA	M3c		
$M3_{crude}$_3	peak @ 5.90 104.03	OPLS-DA	M3a		
$M3_{crude}$_4	peak @ 6.40 75.03	RF and OPLS-DA	M3a		
$M3_{crude}$_5	peak @ 8.52 43.02	OPLS-DA	M3b		
$M3_{crude}$_6	peak @ 8.71 91.98	RF and OPLS-DA	M3a, M3c	$C_4H_8S_2$	505-29-3
$M3_{crude}$_7	peak @ 8.72 60.00*	RF and OPLS-DA	M3a		
$M3_{crude}$_8	peak @ 8.78 200.84	OPLS-DA	M3b		
$M3_{crude}$_9	peak @ 8.96 115.92	OPLS-DA	M3a		
$M3_{crude}$_10	peak @ 9.18 43.02	OPLS-DA	M3a, M3b		
$M3_{crude}$_11	peak @ 9.28 150.03	OPLS-DA	M3b	$C_6H_{11}SCl$ (T) (Δ = 0.7 ppm)	
$M3_{crude}$_12	peak @ 9.35 127.97	OPLS-DA	M3c	$C_4H_{11}O_2SCl$ (T) (Δ = 0.9 ppm)	L
$M3_{crude}$_13	peak @ 10.04 137.02	OPLS-DA	M3c		
$M3_{crude}$_14	peak @ 10.32 46.98	RF and OPLS-DA	M3b		
$M3_{crude}$_15	peak @ 10.36 44.03	RF and OPLS-DA	M3a, M3c		
$M3_{crude}$_16	peak @ 10.64 127.95*	RF and OPLS-DA	M3a, M3b, M3c	$C_5H_{11}S_2Cl$ (T) (Δ = 0.2 ppm)	
$M3_{crude}$_17	peak @ 10.80 41.04	RF and OPLS-DA	M3a		
$M3_{crude}$_18	peak @ 11.53 120.01	RF	M3b		
$M3_{crude}$_19	peak @ 11.63 71.05	OPLS-DA	M3b		
$M3_{crude}$_20	peak @ 12.03 193.93	OPLS-DA	M3a, M3b		
$M3_{crude}$_21	peak @ 12.64 67.05	RF	M3c		
$M3_{crude}$_22	peak @ 12.67 202.02	RF	M3c		
$M3_{crude}$_23	peak @ 13.16 156.03	RF and OPLS-DA	M3c		
$M3_{crude}$_24	peak @ 13.18 155.03	RF and OPLS-DA	M3a, M3c		
$M3_{crude}$_25	peak @ 13.39 93.96	RF	M3a		
$M3_{crude}$_26	peak @ 13.40 59.00	RF	M3a, M3c		
$M3_{crude}$_27	peak @ 13.43 189.94	RF	M3c		
$M3_{crude}$_28	peak @ 13.53 193.96	OPLS-DA	M3c		
$M3_{crude}$_29	peak @ 13.54 140.98	OPLS-DA	M3b		
$M3_{crude}$_30	peak @ 13.55 186.00	OPLS-DA	M3c	$C_6H_{12}SCl_2$ (T) (Δ = 0.3 ppm)	L
$M3_{crude}$_31	peak @ 13.63 171.98	RF	M3b		
$M3_{crude}$_32	peak @ 14.19 170.98	OPLS-DA	M3a		
$M3_{crude}$_33	peak @ 14.49 184.96	OPLS-DA	M3c		
$M3_{crude}$_34	peak @ 14.57 145.04*	OPLS-DA	M3a		
$M3_{crude}$_35	peak @ 14.72 205.16	RF and OPLS-DA	M3a, M3b, M3c		
$M3_{crude}$_36	peak @ 14.75 184.96	OPLS-DA	M3c		
$M3_{crude}$_37	peak @ 14.80 43.02	RF and OPLS-DA	M3b, M3c		
$M3_{crude}$_38	peak @ 14.84 69.07	RF and OPLS-DA	M3a, M3b		
$M3_{crude}$_39	peak @ 15.05 86.02	OPLS-DA	M3b		
$M3_{crude}$_40	peak @ 15.41 89.01	OPLS-DA	M3b		
$M3_{crude}$_41	peak @ 15.52 105.99	OPLS-DA	M3b	$C_5H_{10}S_2Cl_2$ (T) (Δ = - 0.3 ppm)	63869-13-6
$M3_{crude}$_42	peak @ 15.54 240.01	OPLS-DA	M3c		
$M3_{crude}$_43	peak @ 15.59 41.04	RF	M3b		
$M3_{crude}$_44	peak @ 16.39 91.03*	OPLS-DA	M3a, M3c		
$M3_{crude}$_45	peak @ 16.81 60.00	OPLS-DA	M3b		
$M3_{crude}$_46	peak @ 16.92 91.03	OPLS-DA	M3a		
$M3_{crude}$_47	peak @ 16.99 122.00	OPLS-DA	M3b		
$M3_{crude}$_48	peak @ 17.01 123.00	RF	M3b		
$M3_{crude}$_49	peak @ 17.23 215.96	RF and OPLS-DA	M3b	$C_6H_{10}S_2Cl_2$ (T) (Δ = 1.1 ppm)	
$M3_{crude}$_50	peak @ 17.43 116.98	OPLS-DA	M3b		
$M3_{crude}$_51	peak @ 18.10 69.07.1	RF	M3a		
$M3_{crude}$_52	peak @ 18.37 243.06	RF and OPLS-DA	M3a	$C_{13}H_{19}OSCl$ (T) (Δ = 0.6 ppm)	
$M3_{crude}$_53	peak @ 19.24 199.11	OPLS-DA	M3a		
$M3_{crude}$_54	peak @ 19.51 123.00	OPLS-DA	M3c		

*= found in matrix models, T=tentative molecular formula, calculated by HRMS data. Δ= ppm deviation of calculated value from theoretical elemental composition.

Table S4. Most important variables in RF and OPLS-DA $M2_{matrix}$ models separating chlorination methods.

ID number	Peak ID Ret. time and m/z	Correlated to	Best match of molecular formula of fragment
$M2_{matrix}$_1	peak @ 13.29 139.97	R(2,5,8)	$C_2H_5O_3PS$ (Δ = -1.1)/$C_3H_5O_2ClS$ (Δ = -2.4)
$M2_{matrix}$_2	peak @ 20.85 166.99	R(2,5,8)	$C_4H_8O_3PS$ (Δ = 0.3)
$M2_{matrix}$_3	peak @ 17.29 137.02	R(2,5,8)	$C_5H_{10}ClS$ (Δ = 0.3)
$M2_{matrix}$_4	peak @ 15.31 105.05	R(3,6,9)	$C_5H_{10}Cl$ (Δ = 0.4)
$M2_{matrix}$_5	peak @ 13.63 140.98	R(2,5,8)	$C_3H_6O_2ClS$ (Δ = 0.3)/$C_2H_6O_3PS$ (Δ = 1.6)
$M2_{matrix}$_6	peak @ 18.01 159.92	R(3,6,9)	$C_2H_5ClS_3$ (Δ = -0.9)
$M2_{matrix}$_7	peak @ 15.60 123.00	R(2,5,8)	C_4H_8ClS (Δ = 0.2)
$M2_{matrix}$_8	peak @ 16.73 215.96	R(2,5,8)	$C_6H_{10}Cl_2S_2$ (Δ = 1.2)
$M2_{matrix}$_9	peak @ 15.45 105.05	R(3,6,9)	$C_5H_{10}Cl$ (Δ = 0.5)
$M2_{matrix}$_10	peak @ 5.43 60.00	R(2,5,8)	C_2H_4S (Δ = -1.0)
$M2_{matrix}$_11	peak @ 16.36 123.95	R(1,4,7)	$C_2H_4S_3$ (Δ = 2.7)
$M2_{matrix}$_12	peak @17.06 85.01	R(2,5,8)	C_4H_5S (Δ = 0.6)

Δ= ppm deviation of calculated value from theoretical elemental composition.

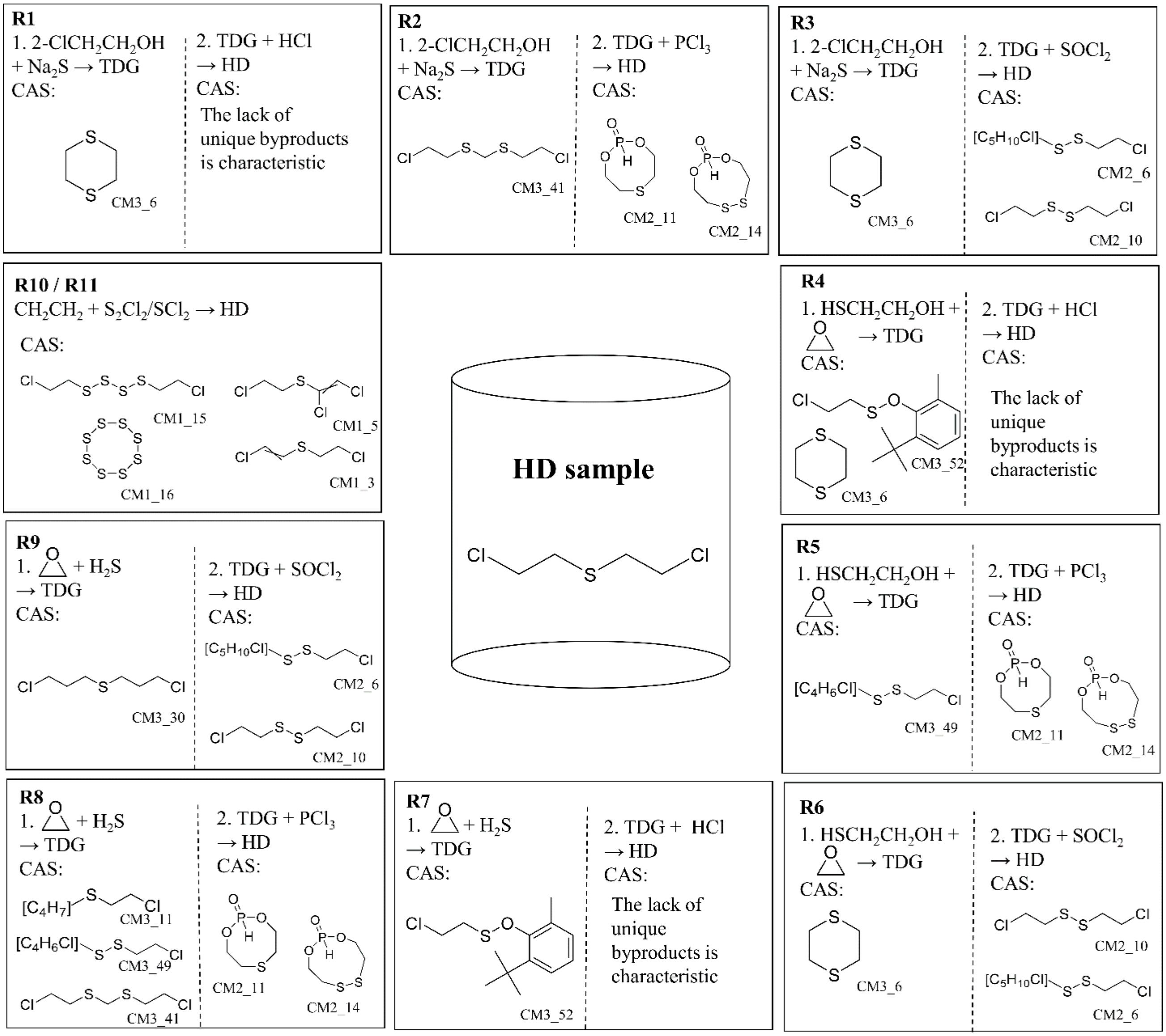

Figure S1. HD synthesis descriptions and representative CAS.

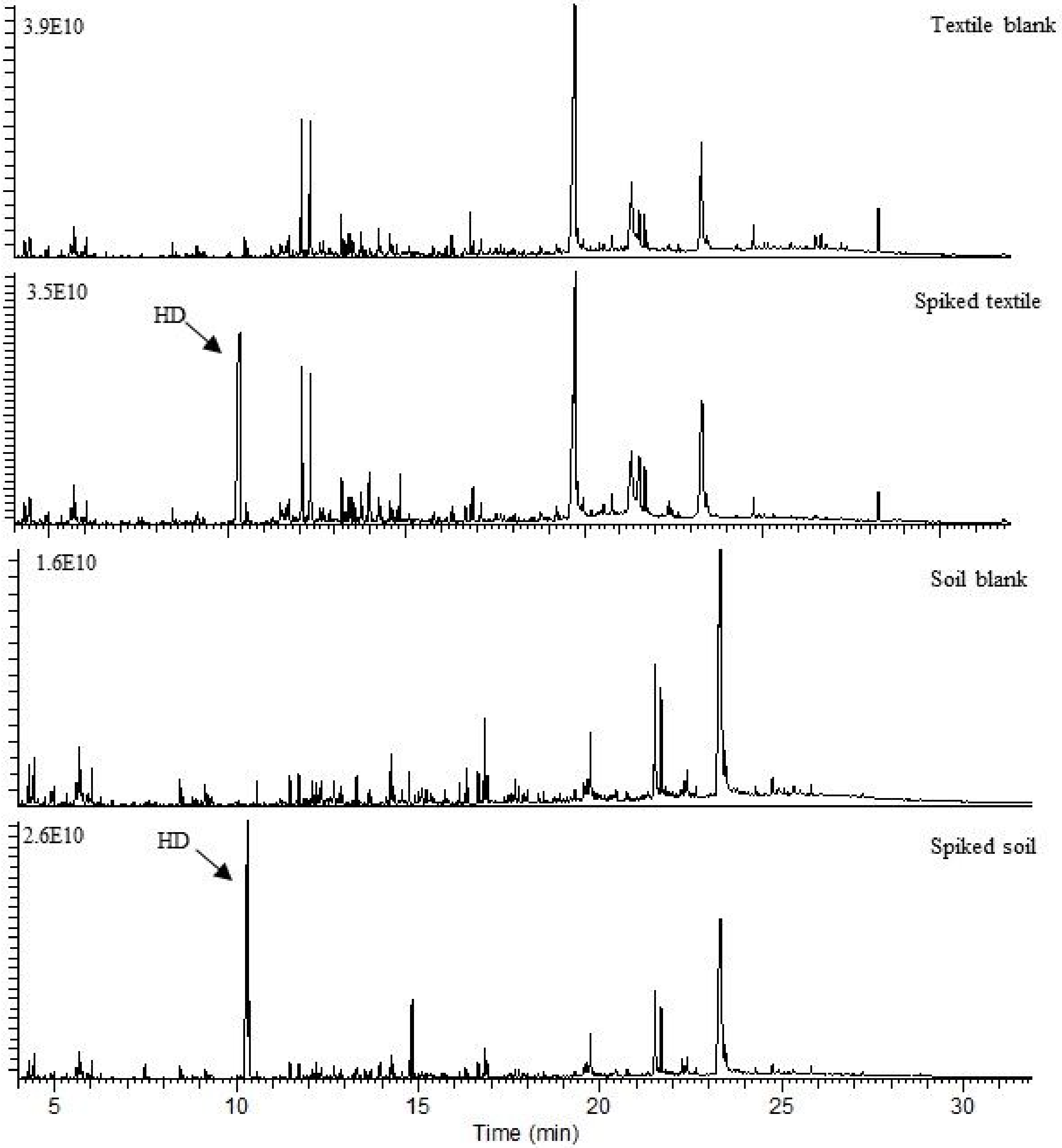

Figure S2. Total ion chromatogram of blank matrix samples and spiked (R8) matrix samples. The HD (*) peak is seen at a retention time of 10.1 minutes.
